# Sustainable Orthopaedic Surgery Residency Training in East Africa: A 10-Year Experience in Kenya

**DOI:** 10.5435/JAAOSGlobal-D-19-00035

**Published:** 2019-07-30

**Authors:** Eric Gokcen

**Affiliations:** From the Department of Orthopedic Surgery and Sports Medicine, Lewis Katz School of Medicine at Temple University, Philadelphia, PA.

## Abstract

Low- and middle-income countries (LMICs) have continued to lag behind high-income countries in all measurable outcomes of health care. As concluded by the World Health Organization during the 2013 Global Forum on Human Resources for Health, an adequate healthcare workforce is mandatory to provide universal health coverage. Despite efforts to increase the numbers of healthcare workers, an extreme deficit in highly trained surgeons remains. Several options exist to provide training for surgeons in LMICs, including local training by local surgeons, sending local surgeons abroad for training, or local training by short-term or long-term visiting surgeons from high-income countries. This article further discusses the benefits and challenges of each option and reviews the 10-year outcomes of the Orthopaedic Surgery Residency Program at the CURE Kenya Hospital in Kijabe, Kenya. The program has graduated nine orthopaedic surgeons who are all practicing in Africa, five of which are full-time attending consultants in residency training programs. An additional eight residents are currently in the program. Sustainable orthopaedic training can be accomplished in LMICs as demonstrated by the ongoing success of the CURE Kenya Orthopaedic Surgery Residency Program. Additional efforts to expand and replicate this model may assist in providing improved access to high-quality universal healthcare in LMICs.

Low- and middle-income countries (LMICs), defined by the World Bank as having a gross national income per capita of $3895 or less,^1^ have continued to lag behind high-income countries (HICs, gross national income per capita greater than $12,055) in all measurable outcomes of health care, despite numerous programs and billions of dollars in aid.^2,3^ Most of the funding and focus have been on high-yield, high-volume programs, such as clean water, immunization, and nutrition, that prevent disease. However, when disease or trauma does occur, the healthcare infrastructure to care for athe disease or injury is typically found lacking. Developing and funding a medical system to treat disease and injury is much costlier and time-consuming. Although immunization, clean water, and good nutrition have nearly immediate, measurable results, the development of a skilled medical system takes years and decades to see results.

Some countries have been applauded for expedited training of many low-level health workers to bring basic health care to communities.^4^ The training has relatively minimal expense, and the trainees are quickly incorporated into the community health system. However, the level of health care provided cannot exceed the level of the highest trained health workers. Community healthcare workers can only provide limited expertise in the care they provide. To raise the standard of care in a community, there needs to be the training and development of experts in health care, namely physicians. Therefore, despite the increased costs and the long-term commitments required, to raise the medical system of LMICs to match or exceed the standards in HICs, it is paramount to invest in training and educating good doctors.^3^

An extreme deficit of physicians remains in LMICs.^2,3,5,6^ In response to this, many countries have expanded their medical schools to increase the numbers of doctors, believing that increased numbers will lead to better care and better access.^7,8^ However, as any physician will testify, finishing medical school does not make a practicing doctor. Certainly, more medical schools are needed; however, graduates must continue with residency training. This is where they really learn how to take care of patients, and for surgeons, how to operate. Little attention has been afforded to the development of residency programs, as it appears that most funding organizations see graduation from medical school as the goal. However, the final goal should be fully trained and competent physicians, ready to care for patients' needs within their specialty.

Within the workforce of physicians are surgeons, and the deficit is also extreme in LMICs.^9,10^ For a time, surgery was not considered essential to the health of cohorts in LMICs, and thus, the training and development of surgeons has been very limited. As Ozgediz et al^10^ has succinctly stated, “The common perception that surgical care is merely a luxury in poor countries must be reconsidered and its essential role in global public health must be acknowledged. Anything less will ensure that the morbidity and mortality endured by millions of people in poor countries unable to access surgical care will continue to remain invisible to the rest of the world.” Fortunately, the focus has slowly begun to change over the past decade, and a growing support is found to increase access for surgery in LMICs.^11,12,13,14^ Indeed, the Copenhagen Consensus of May 2008 considered essential surgery as a potential priority for the poor.^10,13^

In addition, the need for surgeons continues to grow. From 1990 to 2010, a 25% increase has been found in noncommunicable disease with a 13% increase in injuries.^15^ Many of these disease entities require surgery, especially for trauma. As the developing world becomes more industrialized and motor vehicles increase in numbers, the need for trauma care will continue to increase.^16,17^ In addition, trauma typically affects previously healthy persons who are of working age, and these people are often the sole provider for an extended family. Any deaths or resultant disabilities to each of these individuals have a profound negative effect on the independence and well being of a large number of people.^16^

In light of the need for surgeons to provide necessary health care and the current deficit of surgeons, focusing on training an increased number of excellent surgeons is essential.^18^

## Training Surgeons of Excellence

Several options exist in the approach to training surgeons in LMICs including (1) use local surgeons to do the training, (2) send the trainees overseas for training in HICs, (3) send HIC surgeons on short-term trips to train in-country, or (4) send HIC surgeons to teach long term in-country. As one of the few orthopaedic surgeons in Kenya, Mulimba, in his article, Orthopaedic Training in Kenya,^19^ discussed these options and their issues from the point of view of a Kenyan. Benefits and challenges to each option are found, which will be discussed in greater detail later.

Training local physicians with local established surgeons has many benefits. The trainees and the trainers stay in their home country, making this the least costly option. There is no need for the expense of bringing HIC surgeons into the country. However, this system depends on the trainers having been adequately trained in all aspects of orthopaedic surgery. Most orthopaedic surgeons in LMICs have not received advanced training in joint arthroplasty, arthroscopy, and many other procedures. In addition, a limited number of surgeons are found to provide the training, and with their current schedules overloaded, they may have limited availability. Many national fully trained surgeons spend little time at the teaching hospitals but instead focus their time working at the private hospitals where the pay is notably greater. Thus, either the local surgeons are not trained well enough, or there are not enough well-trained surgeons available to develop an effective training program.^19^

LMICs have also sent good candidates to HICs for training, with the hopes that they would return to their home country as highly trained and skilled surgeons. Unfortunately, most of these surgeons end up staying in the HICs and never return to their home countries.^20,21,22^ This so-called brain drain has left the LMICs with fewer good candidates for health care where they are needed the most and, ironically, has increased the HIC level and number of surgeons where they are not needed as much. In the United States, 25% of the physician workforce are international medical graduates, and of which, 60% are from LICs.^22^ In the 1980s, 60% of the trained doctors in Ghana left the country. Not only does the home country lose physicians but it also adds cost to the economy. According to 1996 estimate, for every professional who left an African country, the economic loss to that country was $184,000. In a 2000 estimate, South Africa lost over 600 graduates to New Zealand equal to $37 million.^20^ Some of these surgeons do return to their homes but, owing to a lack of funding and equipment, have been unable to have a lasting effect on training residents. In addition, they are often the only HIC-trained surgeon in their department, which can create conflict with the locally trained surgeons in approaches to training. Ultimately, this approach to improving the level of health care in LMICs has had little positive effect and may have even worsened overall health care in some circumstances.^20,21,22^

Short-term mission trips from HICs have been ongoing for many years. Initially begun by medical missionaries, having different orthopaedic societies sending humanitarian teams to LMICs for short-term trips is now common. Typically, they do many surgeries and incorporate some degree of teaching into the trip. These trips can be very successful in providing necessary surgeries for the local people that would otherwise be unavailable, such as joint arthroplasty and arthroscopic procedures. However, several issues also present. Although a week or two of lectures and seeing specific procedures is educational, providing adequate teaching for the local surgeons to actually begin doing the procedures falls far short. A surgeon cannot learn how to do a hip arthroplasty from a week or two of lectures and surgical observation. In addition, concerns are always about patient follow-up. What happens if a patient needs advanced surgical intervention after the team leaves?

At times, well-intentioned visiting surgical teams will go to countries where national surgeons are found doing the same surgeries as the visiting team. As a result, the visiting team is actually competing with the local surgeons for cases, thus having a negative economic effect on the local surgeons. Typically, these mission teams do not charge for their services and provide charity or nearly free surgery, which makes it even more of a negative effect on the local surgeons' ability to get paying patients. Those getting the “free” surgeries are not always the poorest, but usually the best connected.

Short-term medical mission trips can still be beneficial if the abovementioned issues are evaluated and guidelines are followed, as established by Grimes et al.^23^ However, as popular as these trips have become, in and of themselves, they do not provide a long-term sustainable model for improving surgical healthcare in LMICs.

Long-term HIC surgeons have notable advantages over the other options but also come with notable challenges. Having a HIC surgeon stay long term allows for the development of deeper relationships and understanding with the local surgeon trainees, and provides an avenue of extended education of the local trainees by a well-trained surgeon. This process has the ability to mimic the current training in HICs by providing a structured curriculum covering all aspects of surgical training.

Adequately trained surgeons are available for not only teaching but also providing much needed surgeries that would otherwise be unavailable. The surgeon would be able to provide all postoperative care along with treating any unexpected complications appropriately, which would also allow surgical trainees the ability to experience and learn how to treat any surgical complications. Complete surgical training involves not only how to diagnose and treat surgical conditions but also how to manage them postoperatively, with or without complications.

In addition, the HIC surgeon typically has connections with industry and has the ability to obtain modern equipment and supplies that are otherwise not available in the host country, such as arthroscopy equipment and joint arthroplasty implants. They can often obtain the equipment at a reduced rate, making it more feasible and cost effective.

The challenges of a long-term HIC surgeon are obvious: the surgeon would need to leave his home country, including family and friends, along with the negative economic effect of leaving a thriving practice. Although this condition does not sound feasible, surgeons who are willing to go for extended periods to teach in LMICs are found. These surgeons need to be supported to encourage them to go and to stay. Their home practice may be able to keep their position for when they come home for short or intermediate stays, so that the surgeon can generate some income and maintain CME and licensing standards. These home practices could also provide necessary medical insurance and the other benefits the surgeons have while at home. In addition, outside funding is necessary to help mitigate some of the costs incurred by the long-term surgeon.

A system to encourage and support HIC surgeons to go long term to LMICs was developed by CURE International when they opened their first hospital in Kenya in 1998. CURE provided the framework for a surgeon to go and subsequently started residency training programs. In addition, they were able to provide up-to-date facilities and equipment, which helped the local people obtain access to more modern procedures and also allowed the trainees to be trained on newer, more modern surgical techniques.

## The CURE Kenya Experience

CURE International was founded by Scott Harrison, MD, and his wife Sally Harrison in 1996 after recognizing the need for improved orthopaedic care and education in the developing world. CURE built and opened its first hospital in Kenya in 1998 and has since opened hospitals in several countries around the world focusing on surgical subspecialty care and training.

CURE's focus on education led to the initiation of an Orthopaedic Surgery Residency in 2007 at the CURE Kenya Hospital in Kijabe, Kenya, in conjunction with the Kijabe Mission Hospital and Moi University in Eldoret, Kenya. The program received official accreditation by College of Surgeons of East, Central, and Southern Africa (COSECSA).

COSECSA was founded in 1999 by a group of surgeons and with support from the Royal College of Surgeons of Edinburgh. They realized that good education required input from other good surgeons but ultimately would require growth and governance by national surgeons. In addition, COSECSA developed standards of education that mirrored HIC education, requiring specific goals and objectives, which includes a wide variety of elective orthopaedics in both children and adults, trauma, preclinical, and other surgical disciplines such as head and chest trauma, anesthesia, and caesarian sections. The training includes didactic, clinical, and online learning, in addition to attendance of several training conferences each year. Residents must maintain a detailed logbook of their cases, which is reviewed by the credentials committee before their final examinations at the end of their final year. No specific case numbers are noted by COSECSA for graduation, but it is taken into consideration when being evaluated to be able to sit for the final oral examination. Residents are required to pass rigorous written examinations and a final oral examination to complete their training, much like board examinations in HICs. The length of training is similar to HICs with orthopaedics requiring 5 years of training after completing medical school.^19^ Acceptance into the residency program requires successful completion of an MD program accredited by the host country. Applications are reviewed and applicants selected based on the strength of their application and interviews.

The CURE Kenya residency program initially began with three full-time faculty including two HIC orthopaedic surgeons, both board certified in the United States, and one national Kenyan orthopaedic surgeon who had been under the tutelage of one of the HIC surgeons for many years. He had received an MMed(surg) degree in Uganda along with his initial orthopaedic training there. As the program matured, eventually the HIC surgeons were able to leave and the Kenyan graduates of the program became the faculty instructors. The residents are now fully trained in most of the subspecialties in orthopaedic surgery, including arthroscopy, adult joint reconstruction, spine surgery, hand surgery, pediatric surgery, and trauma. HIC surgeons now come for short-term trips to assist in high-level education, both in and out of the operating rooms.

The CURE Kenya residency has continued to grow and maintain high standards of educational excellence. The current residents include eight physicians from Kenya and one from the Democratic Republic of Congo. Nine residents have graduated from the program thus far and are practicing orthopaedic surgeons in their home countries. Seven are in Kenya, two in Ethiopia, and one in Cameroon. Three of the graduates are full-time attending orthopaedic surgeons teaching in Kenya. The two residents from Ethiopia who completed the program have been full-time attending pediatric orthopaedic surgeons at the CURE Ethiopia Hospital for over 5 years and are actively involved in the training of Ethiopian orthopaedic residents through the national Ethiopian residency program.

**Figure 1:**
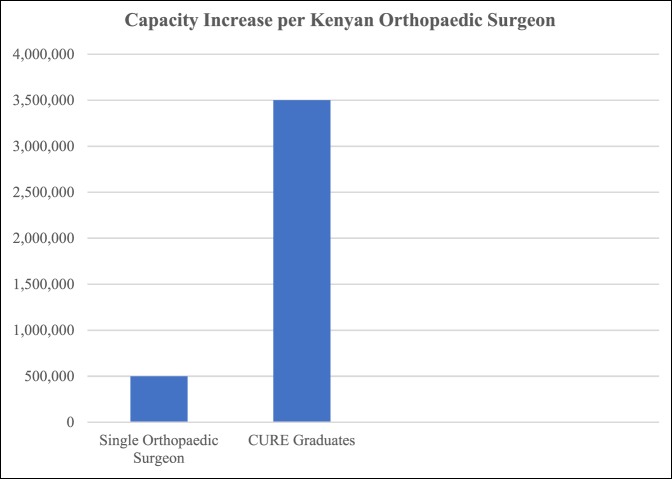
Bar graph showing cohort capacity for a single Kenyan orthopaedic surgeon and for the total number of current Kenyan graduates (seven) from the CURE Orthopaedic Residency Program.

Although these numbers appear small, one must realize that only 86 orthopaedic surgeons are present currently in Kenya for a cohort of 45 million. These 86 orthopaedic surgeons have wide variability in their training, much as HICs did in the infancy of orthopaedic surgery.^19^ Each additional orthopaedic surgeon has a notable effect on improving access for patients (Figure 1). In addition, those who continue on as instructors can result in exponential growth in surgeon numbers.

The success in the program comes from several points. First, CURE International, a US nongovernment organization that constructs and manages the hospitals, places a high level of importance on education and training. In addition to orthopaedics, there is training for anesthesia, nursing, and other skilled healthcare workers. CURE secures funding for the training, which is supported by individual donations and grants. Without outside funding, this level of training would not be possible.

Second, CURE is able to recruit long-term educators from HICs who live and work in the host country, usually for several years at a time, which is a critical part of the success of the program. The long-term educators are board-certified orthopaedic surgeons who leave their practices in the HICs to bring their knowledge and skill set to an LMIC and teach. Staying for several years allows for deeper and more consistent training. Residents are held to the standards. Goals and objectives are maintained and attained. Leadership, ethics, and professionalism are instilled. All these points require an extensive time commitment from the educator and are only effective if the educator is present long term.

Finally, a program is most likely to become sustainable when the trained nationals become the educators. CURE Kenya is now completely run by Kenyans. Any orthopaedic surgeon who visits the hospital will be impressed by the level of education of the residents. Many of the graduates are actively training orthopaedic surgeons in their African home countries.

A successful program such as this also has its challenges. Funding is difficult, but not impossible. Once a program is successful, it opens the doors for others to support its mission. Ultimately, the government needs to provide some level of funding, as is done in HICs.

The largest challenge in building the program is finding the long-term educators. Surgeons who are willing to leave their families and the comfort (and income) of their homes to go abroad to live long term in an austere environment are not easy to find. Many younger surgeons are burdened by student loan debt and cannot afford to leave a lucrative practice. Some mission organizations have surgeons interested in going, but the numbers are few. Another option is to have HIC universities partner with programs in LMICs and provide an ongoing rotation of surgeons to go abroad and teach.^24,25,26^ The universities also have better access to funding and may be able to provide assistance in supporting the expenses of the residency training program.

## The Future

The CURE program has been instituted in several LMICs and continues to be successful. Further growth of their program or the development of similar programs in other organizations would be optimal. As the numbers of well-trained surgeons in LMICs continues to increase, then the approach to training will need to change. These well-trained surgeons can become the local in-country trainers, leading to less expense and potentially an exponential growth in the numbers of LMIC surgeons.^27^ A similar program to CURE's program was instituted in Malawi with similar success, using funding from the Norwegian government and teaching from the Haukeland University Hospital and the University of North Carolina.^24^ In addition, CURE has other orthopaedic surgery residency programs in Ethiopia, Malawi in conjunction with the Queen Elizabeth Central Hospital, and Zambia, with a neurosurgery program in Uganda.

As the residency programs mature, long-term HIC surgeons will become less and less necessary. Instead, HIC short-term trips will become much more effective, as they will be able to teach more advanced surgical techniques to well-trained established surgeons, who, in turn, can teach others in their country. Short-term trips are better for educating surgeons who already have basic training in their specialty. For example, learning how to do a new approach for a hip arthroplasty could be learned in a week or two if the learner already knows and does hip arthroplasties. In addition, visiting short-term HIC surgeons who focus on education rather than performing large numbers of surgeries will also avoid the economic competition with the local surgeons for cases. There will no longer be a need for HIC surgeons to bring in donated equipment and supplies. The increased demand for these will be driven by the local surgeons, and private companies will meet the need. In fact, as a result of the improved training in Kenya, the orthopaedic industry now has distributorships locally providing advanced implants and equipment.

One of the other benefits of training LMIC surgeons in-country is that after completing their training, they are less likely to leave their country for HICs. Graduation from a COSECSA accredited program is acceptable to African countries, but not in HICs. If a graduate surgeon wanted to move to a HIC, he would be required to repeat a full 5 years of training, which is not appealing to most. Also, they have developed roots and a reputation in their home country and are more likely to be satisfied staying home.

After LMIC surgeons who have completed their in-country residency, they can be invited for more advanced training in HICs. One of the surgeons who completed the Kenya residency is now completing a prestigious pediatric orthopaedic fellowship in a HIC and will return back to his home country on completion. He is an attending surgeon at a teaching hospital in his home country, and this further advanced training will greatly benefit the training of the in-country residents. Acceptance to a HIC-advanced training program is of course at the discretion of the host program. Three surgeons have completed their in-country residency and subsequently completed further subspecialty training in pediatric orthopaedics and sports medicine, primarily in the United States.

Of course, this system of training, as with any training, requires funding. Most development global health programs prefer to see large numbers of trainees with lower expenses. Surgeons are small numbers with higher costs, but ultimately, the national level of quality care will be determined by these physicians. Highly trained surgeons will demand higher quality patient care. Therefore, if the goal of global surgical health care is to elevate the level of care and improve access, funding for surgeon training is mandatory.

## Conclusion

Sustainable orthopaedic surgery training programs in LMICs are vital to improve the health care in these countries. As demonstrated by the success of the CURE Kenya Orthopaedic Surgery Residency Program, this can be accomplished through the commitment of funding and long-term educators in partnership with accreditation institutions to ultimately produce highly skilled, compassionate, ethical, and professional surgeons. These highly trained surgeons can then become the educators in their home countries, placing less reliance on HICs for education. HIC surgeons can continue to contribute to the education in LMICs through inviting LMIC surgeons to HIC fellowship programs and through short-term, educationally focused trips.
